# Parental Perspectives on Early Life Screening and Genetic Testing for ASD: A Systematic Review

**DOI:** 10.1007/s10803-023-06231-z

**Published:** 2024-02-14

**Authors:** Katerina Dounavi, Meral Koldas

**Affiliations:** 1 School of Social Sciences, Education & Social Work, Queen’s University of Belfast, 20 College Green, Belfast, BT7 1LN Northern Ireland United Kingdom; 2 Present Address: Integrated Center for Autism Spectrum Disorders (iCASD), Munroe-Meyer Institute, University of Nebraska Medical Centre, Omaha, USA

**Keywords:** Autism, Parental perspectives, Early life screening, Genetic testing

## Abstract

Autism Spectrum Disorder (ASD) is a prevalent neurodevelopmental condition for which no prenatal or early life screening tests exist. Early life recognition of ASD is key to accessing behavioral intervention when brain plasticity is at its peak. The purpose of our study was to systematically review the literature researching parental perspectives around early life screening for autism and specifically genetic testing. A total of 30 studies were included and coded against the following variables: parental characteristics, child characteristics, research design, data collection and data analysis methods, type of early screening, and parental perspectives towards early life screening and genetic testing. The outcomes of the review showed that caregivers need more knowledge about ASD genetic testing, they are in general in favor of early life screening, and they prefer to access ASD genetic testing and early behavioral intervention as early as possible. As emerging genetic tests are likely to increase diagnostic accuracy for ASD in the near future, it is of paramount importance for research and practice to embrace parental needs and preferences. Healthcare providers can be pivotal in empowering parents to make informed decisions through clear, compassionate communication and counseling. Future research should seek to fill in an essential gap in the literature, which is to capture parental views from a diverse population.

Autism spectrum disorder (ASD) is a neurodevelopmental condition characterized by limitations in social communication, as well as the presence of repetitive and restricted behaviors and interests (American Psychiatric Association, 2013). The reported prevalence of ASD has significantly increased over the past decade, with the latest reports showing a prevalence of 1 in 36 children (Maenner et al., 2023). ASD often manifests in the first two years of life, although it is most often diagnosed at a later age (Zwaigenbaum & Penner, 2018). The rapid increase in the number of children diagnosed with ASD and the need to support them is exerting a greater demand on experts and institutions to provide early and reliable identification (Makino et al., 2021).

Early identification of ASD is crucial to ensuring that individuals with ASD have access to evidence-based interventions that will enable them to reach their full potential and improve long-term outcomes (Tanner & Dounavi, 2020; Zwaigenbaum et al., 2015). The goal of early identification is not only to confirm an ASD diagnosis but also to help individuals and their families understand the diagnosis (Sher-Censor et al., 2017), direct caregivers towards appropriate services (Abbott et al., 2013; Zwaigenbaum & Penner, 2018), detect any co-occurring conditions that require treatment (King et al., 2009), and reduce the anxiety that many families experience prior to ASD diagnosis (DesChamps et al., 2020; Rogers et al., 2016; Voliovitch et al., 2021). However, the time between the onset of core autism symptoms and an ASD diagnosis is still unacceptably long. For instance, a study by Penner et al. (2018) in Canada found that the average waiting time for an ASD diagnosis was 7 months (interquartile range 4–12 months). In the UK, 85% of children wait longer than the recommended 13 weeks with the average waiting time being just under a year, 28% waiting between 1 and 2 years, and 20% waiting between 2 and 3 years (Ambitious about Autism, 2021; House of Commons, 2023); post-pandemic data reported by the National Health System are even worse (NHS, 2023). This is albeit the National Institute for Health and Care Excellence stating that no family should wait more than 13 weeks for an autism assessment and 18 weeks for treatment to start (NICE, 2011). European data are equally daunting (Magán-Maganto et al., 2017).

Currently, the diagnosis of ASD relies on behavioral observations, often combined with the use of standardized instruments, such as the gold-standard ADOS-2 (Lord et al., 2012). In the last decade, early life ASD identification has gained force in research as an important area to explore and a few new tools have emerged that can help professionals identify early signs of ASD before the age of 2 years (Tanner & Dounavi, 2021). Such tools include behavioral and medical/genetic screening tests that can detect signs of autism in young children or even before birth. Behavioral screening tests are used to evaluate the child's social interaction, communication, play, and sensory sensitivity (Johnson & Myers, 2007). Behavioral assessments might take the form of questionnaires, observations, interviews, and standardized scales such as the Childhood Autism Rating Scale (CARS; Chlebowski et al., 2010) or The Modified Checklist for Autism in Toddlers (M-CHAT; Robins et al., 2014). Medical tests have been used to rule out or identify any potential genetic or medical causes of ASD, such as chromosomal abnormalities, metabolic disorders, or brain abnormalities (Zhao et al., 2021a, 2021b). Medical tests may take the form of blood tests, urine tests, brain scans, or genetic tests, with the latter including chromosomal microarray analysis, G-banded karyotyping, and fragile X testing. Both behavioral and medical tests are important as they can provide a comprehensive assessment and lead to a reliable diagnosis of ASD that will inform support or treatment options for the individual and their family.

When examining more closely the role of medical tests for ASD and despite the availability of standardized screening tests for disorders like Down’s, Edward’s, and Patau’s syndromes for pregnant women (Crombag et al., 2014), similar genetic tests are not available for ASD. Instead, researchers have focused on identifying behavioral indicators for autism that emerge as early as at 6 months of age to address this gap (Tanner & Dounavi, 2021). Recent developments in biomarker research also hold promise for early detection of ASD (Hnoonual et al., 2017; Xu et al., 2016), although further research is necessary for the field to progress.

Research on the genetic origins of ASD has led to the identification of common genetic variants, which explain a portion of all cases of ASD. In addition to identifying variants, ongoing genetic research has focused on the identification of signalling pathways and altered functions (Fang et al., 2023; Qiu et al., 2022; Rodriguez-Gomez et al., 2021; Schaefer & Mendelsohn, 2008). Albeit important discoveries, a large genetic and clinical heterogeneity remains among people with ASD. However, as our knowledge of the genetic etiology of ASD has advanced, recommendations from authorities have increasingly embraced genetic testing as a means to understand the nature of ASD and enable individuals to access treatment early. For example, the American College of Medical Genetics and Genomics recommends that genetic evaluation should be offered to all persons and families with ASD (Schaefer & Mendelsohn, 2008), while the Centers for Disease Control and Prevention support an individualised approach to genetic testing (Centers for Disease Control & Prevention, 2022), yet only about one third of this population has ever undergone genetic testing (Zebolsky et al., 2020). To ensure that research and clinical practice align with the needs and preferences of families, it is important to consider parental perspectives as we work towards standardizing early life ASD screening and genetic testing (Reiff et al., 2015).

Notwithstanding the importance of involving parents in decisions around early life testing for ASD in their offsprings, there is limited research on parental perspectives and experiences around early life ASD screening. While some existing reviews have focused on parental perspectives on the diagnostic process (Makino et al., 2021; McCrimmon & Gray, 2021; Naicker et al., 2023), no reviews have specifically addressed the topic of early life ASD screening, including prenatal and early life tests, which may have important implications for parental decision-making. Therefore, the aim of the present systematic review is to identify and synthesize the literature on parental attitudes towards early life genetic testing for ASD and to determine the acceptability and usefulness of such testing.

## Method

### Searches

Searches were conducted in January and February 2023 in three databases: PsychINFO, MEDLINE and PubMed, filtering by title. The string of employed search keywords was “Autism OR ASD OR PDD OR developmental disorder OR autistic” AND “screen* OR test* OR diagnos*” AND “parent* OR caregiver*”.

### Inclusion/Exclusion Criteria

Studies were included if they: (a) yielded original raw data, (b) focused on children with a likelihood for ASD, PDD or developmental delays and their parents or on children with a confirmed diagnosis of ASD, PDD or developmental delays and their parent, and (c) explored parent perspectives around early life (before 24 months) behavioral screening or medical testing for autism, and (d) publication was a peer-reviewed journal article.

Exclusion criteria were: (a) study was a narrative case study, secondary analysis, systematic review or meta-analysis yielding no original data, (b) study focused on children with a likelihood for or diagnosis of a condition other than ASD, PDD or developmental delays or did not involve their parents, (c) study explored later life diagnosis (e.g., diagnostic assessment after the age of 2 years), and (d) publication was not a peer-reviewed journal article (such as conference abstracts, theses, or book chapters).

### Determining Eligibility Procedure

Search results were first scanned, and duplicates were removed. The remaining studies were then merged into a single database and assessed for eligibility against inclusion and exclusion criteria first based on their title and abstract and then based on their full text. Out of 38 studies (10%) cross scanned for eligibility based on their titles and abstracts, an agreement was reached on 28 (74%). A consensus was reached for the remaining 10 (26%) through discussion. At full-text screening, the first author crossed screened 12 out of 53 studies (23%); agreement on the eligibility cross-screening stood at 100%.

**Fig. 1:**
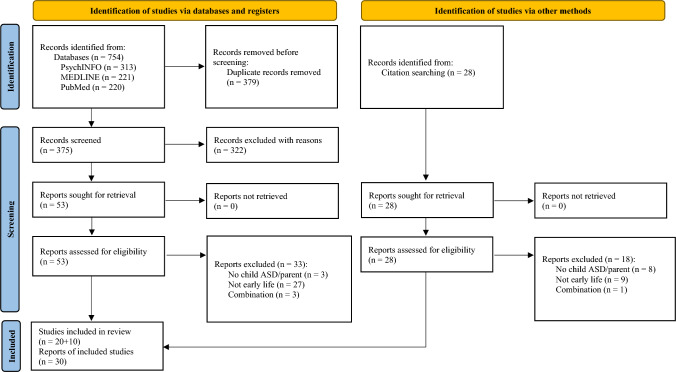
PRISMA flowchart showing identification, screening, and study eligibility process

Upon full-text screening, the second author hand-searched eligible studies to identify additional relevant peer-reviewed journal articles, which were subsequently screened based on their full text (Fig. 1).

### Coding

Data extracted from studies were coded against the following categories:

- Study details: authors, article title, year, DOI, journal name, and country of origin
- Parent/caregiver characteristics: number of participants, caregivers’ gender, participant mean age
- Parent/caregiver demographics: ethnic background, education, income level, spoken language, and family history of autism
- Children participants: diagnosis, an only child with ASD or number of sibling with ASD in the family, age of the child when the study was conducted, and age of the child when genetic testing conducted
- Study research design: qualitative, quantitative, mixed methods
- Data collection modality: phone call, video call, in-person
- Type of early screening: behavioral screening tool, genetic/medical testing
- Data analysis: descriptive statistics, inferential statistics, thematic analysis, etc.
- Parental perspectives towards genetic testing: emerging themes

The second author coded all studies and the first author independently cross-coded a randomly selected 17% (n = 5) of studies. The inter-coder agreement was 100%. After cross coding these five studies, a discussion took place to ensure a common approach and the same level of detail were adopted when coding the remainder of the studies. Finally, a narrative synthesis of findings was produced.

Emerging themes were drafted by the second author after carefully reviewing eligible studies one by one. Themes were then critically revised by the first author to ensure clarity and saturation, and further validated as unique standalone themes (i.e., no overlap between themes) through repeated discussions between both authors (Petruccelli et al., 2022).

To meaningfully synthesize results in positive, negative, or mixed parental attitudes toward genetic testing, we coded outcomes of a study as positive if 75% or more of included participants reported positive attitudes; we coded them as negative if 25% or less of participants voiced positive attitudes; and we coded results as mixed if between 25 and 75% of participants expressed positive attitudes. Four studies were coded as positive, while 26 reported mixed results. None of the studies was classified as negative.

## Results

### Study Distribution Across Time, Journal, and Place

**Fig. 2:**
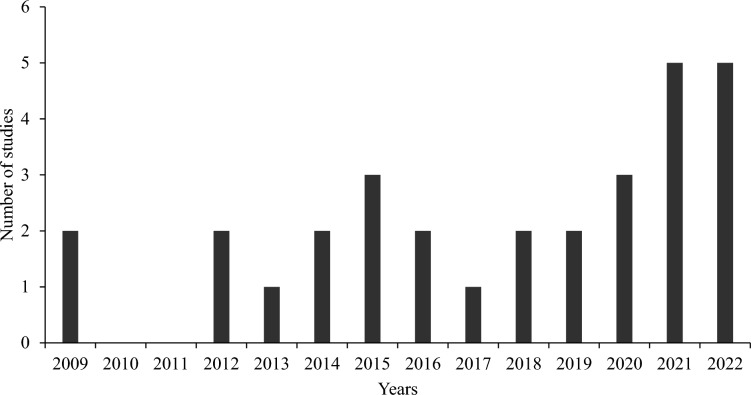
Study distribution across years

A total of 30 studies met our inclusion criteria, with publications spanning the years 2009 to 2022 and showing an increasing trend (Fig. 2). Studies were published in 18 different journals, with most appearing in the *Journal of Autism and Developmental Disorders* (n = 7), followed by *Clinical Genetics* (n = 3), and the *International Journal of Environmental Research and Public Health* (n = 3). Main characteristics and employed research methods of eligible studies together with key outcomes are presented in Table 1. Parent and child characteristics, as presented in included studies, can be found included as Supplementary Information.

**Table 1:** Study main characteristics, employed methods and key outcomes

Study		Research method	Data collection modality					Early screening type		Parental attitudes
Authors	Country		Phone	Video	Online survey	Paper and pencil survey by post	In-person	Behavioral	Genetic	
Amiet et al. (2014)	France USA	Quantitative			X				X	Mixed
Ayhan et al. (2021)	Türkiye (Turkey)	Quantitative			X		X		X Genetic test during pregnancy	Positive
Chen et al. (2013)	USA	Qualitative	X n = 16	X n = 5			X n = 21		X	Mixed
Chen et al. (2015b)	USA	Qualitative					X		X Prenatal genetic testing (PGT)	Mixed
Chen et al. (2015a)	Taiwan	Qualitative					X		X	Positive
Chen et al. (2020)	Taiwan	Quantitative				X	X		Prenatal genetic testing (PGT)	Mixed
Cleary et al. (2022)	Australia	Qualitative					X	X	Finding out the sex of their baby during pregnancy prompted reflection on the possible challenges (i.e., given ASD is more common in boys)	Mixed
Girarelli & Reiff (2015)	USA	Qualitative	X						Chromosomal microarray analysis (CMA)	Mixed
Hendel et al. (2021)	Israel	Quantitative	X						Genetic evaluation includes a test for fragile X syndrome and a chromosomal microarray analysis (CMA) for the detection of copy number variation (CNV) associated with ASD	Mixed
Lemay et al. (2021)	Canada	Mixed	X		X n = 1			X		Mixed
Li et al. (2022)	Taiwan	Quantitative				X			X	Mixed
Lucas et al. (2022)	USA	Quantitative			X n = 24		X n = 30		Chromosome microarray, Fragile-X, single gene or gene panel testing, or WES	Mixed
May et al. (2012)	USA	Quantitative			X				Monoamine oxidase A (MAO-A)	Mixed
Narcisa et al. (2013)	USA	Quantitative			X				X	Positive
Navot et al. (2016)	USA	Qualitative					X	X	X	Mixed
Petruccelli et al. (2022)	USA	Qualitative	X				X	X		Mixed
Reiger et al. (2009)	Canada	Quantitative				X			Array genomic hybridization (AGH)	Mixed
Reiff et al. (2017)	USA	Mixed	X		X				Chromosomal microarray analysis (CMA)	Mixed
Reiff et al. (2015)	USA	Mixed	X		X	X			Chromosomal microarray analysis (CMA)	Mixed
Selkirk et al. (2009)	USA	Mixed			X				X	Mixed
Wagner et al. (2020)	USA	Quantitative					X		Genetic/epigenetic testing for ASD	Positive
Xu et al. (2016)	USA	Qualitative					X		Chromosomal microarray analysis (CMA)	Mixed
Xu et al. (2018)	USA	Quantitative			X		X		Chromosomal microarray analysis (CMA)	Mixed
Zebolsky et al. (2020)	USA	Quantitative			X				X	Mixed
Zhang et al. (2021)	Taiwan	Qualitative					X		X	Mixed
Zhao et al. (2019a)	USA	Quantitative			X				X	Mixed
Zhao et al. (2019b)	USA	Quantitative			X				X	Mixed
Zhao et al. (2021a)	USA	Quantitative			X				X	Mixed
Zhao et al. (2021b)	USA	Quantitative			X				X	Mixed
Zhao et al. (2018)	Taiwan	Qualitative					X		Prenatal genetic testing (PGT) for ASD	Mixed

In terms of the country where research took place (Table 1), the majority of the studies were conducted in the US (n = 20), followed by Taiwan (n = 5; Chen et al., 2015a, 2015b; Chen et al., 2020; Li et al., 2022; Zhang et al., 2021; Zhao et al., 2018), Canada (n = 2; Lemay et al., 2021; Regier et al., 2009), Australia (n = 1; Cleary et al., 2022), France (n = 1; Amiet et al., 2014), Israel (n = 1; Hendel et al., 2021), and Türkiye (Turkey; n = 1; Ayhan et al., 2021). One of the studies was conducted in two countries and was reported as taking place in both France and the US (Amiet et al., 2014).

### Parent/Caregiver Participant Characteristics

A total of 6167 parents or caregivers of at least one child with ASD were included in our synthesis (Supplementary Information). Other reported participants were pediatricians (n = 10), parents of typically developing children (n = 10), and intervention providers (n = 51).

Out of the 30 studies included in our review, 27 reported participants’ gender. The way gender was reported varied between studies, with 15 reporting it in absolute numbers, 8 reporting a percentage that could not be converted in absolute numbers for inclusion in our synthesis, and four reporting it as either female or male, again without providing a total number of participants. Based on data extracted from the 15 studies that reported numbers, 2122 participants were female and 388 were male.

Only 20 studies reported data on participants’ age, with 15 studies reporting a mean age and five studies reporting a percentage of participants belonging to each predefined age range. The majority of participants’ mean age (n = 9 studies) was between 40–45 (Chen et al., 2015a, 2015b; Giarelli & Reiff, 2015; Regier et al., 2009; Reiff et al., 2017; Zhao et al., 2018, 2019a, 2019b, 2021a, 2021b), followed by the age range of 35–40 (n = 5 studies; (Chen et al., 2020; Hendel et al., 2021; Li et al., 2022; Xu et al., 2016, 2018), and one study reporting a mean age of 34.7 years (Wagner et al., 2020). From the five studies that reported a percentage of parents for each age range, the most prevalent age ranges reported were 36–45 (Amiet et al., 2014), > 30 (May et al., 2012), 36–45 (Narcisa et al., 2013), 30–39 (Reiff et al., 2015), and 40–44 (Selkirk et al., 2009).

Similarly, only 20 studies reported data on participants’ ethnicity. Of these, 12 reported participants’ ethnic background in numbers (Chen et al., 2015a, 2015b; Giarelli & Reiff, 2015; Hendel et al., 2021; Lucas et al., 2022; Petruccelli et al., 2022; Reiff et al., 2015; Selkirk et al., 2009; Wagner et al., 2020; Xu et al., 2016; Zebolsky et al., 2020; Zhao et al., 2019a, 2019b), while eight studies reported it as a percentage. From these 12 studies, five specific ethnic groups were reported and an additional “other ethnicity” category. Participants were predominantly identified as White (n = 1639), followed by Asian (n = 279), Hispanic (n = 146), African American (n = 81), and mixed (n = 48); 95 were identified as of “other ethnicity”. In the eight studies reporting ethnicity as a percentage of people belonging in each predefined category, participants were identified predominantly as “White” (Chen et al., 2013), Caucasian (May et al., 2012), Caucasian non-Hispanic (Narcisa et al., 2013), “from varied racial backgrounds with 36% belonging to a minority” (Navot et al., 2016), “White” (Reiff et al., 2017), “Caucasian” (Xu et al., 2018), and “White” (Zhao et al., 2021a, 2021b).

Participants’ religion was reported in nine studies, six of which reported it as the number of participants from a specific religious background (Chen et al., 2015a, 2015b; Hendel et al., 2021; Li et al., 2022; Zhao et al., 2018, 2019a, 2019b) and three as a percentage (Chen et al., 2015a, 2015b, 2020; Zhao et al., 2021a, 2021b). Most participants identified as Christian (n = 752), with the following most reported religions being Buddhism (n = 141), Folk religion (n = 141), Judaism (n = 82), believing in more than two religions (n = 59), and Taoism (n = 10). A total of 152 parents reported following another religion and 260 reported having no religious preference. From the three studies reporting religion in percentages, the predominant religions were Buddhism or Folk religion (Chen et al., 2020) and non-Catholic Christianism (Chen et al., 2015a, 2015b; Zhao et al., 2021a, 2021b).

The participants’ educational background was reported in 25 studies, in 14 of which as a number, in nine as a percentage, and in two with a summarizing statement or value such as “*Mothers tended to be well-educated, all of them having some amount of post-high school education*” (Navot et al., 2016) or “mothers’ and fathers’ median education: 12 years” (Hendel et al., 2021). Across studies reporting education in numbers, most participants had completed a graduate or university course (n = 2611), followed by high school (n = 1179), and postgraduate courses (n = 614). In addition, 501 participants had not completed high school. In studies reporting the percentage of participants who had completed each educational level, again, college/graduate/university was the most commonly reported category, with the exception of one study (Chen et al., 2020) in which two thirds of participants had below college education, with about one third only reporting having completed college or above.

When examining participants’ income level, we identified 17 studies providing data in the form of number/percentage of parents pertaining to each income category or as a summary of the sample’s socioeconomic level. Most studies reported income level in USD, so we have adopted USD in our results. In studies reporting income using a number (n = 7), the majority of participants based in the US (n = 691) reported an annual household income of $75–100 K, followed by $25–50 K (n = 448), $50–75 K (n = 330), and less than $25 K (n = 264). An income of more than $100 K was reported by 46 participants. Taiwanese participants reported NT$600 K (n = 172) as the predominant income level, followed by NT$600 K-1000 K (n = 199), and less than NT$1000 K (n = 122) (Chen et al., 2015a, 2015b; Li et al., 2022; Zhao et al., 2018). Studies reporting percentages confirmed that most participants based in the US had an income of $75 K or above (Chen et al., 2015a, 2015b; Zhao et al., 2021a, 2021b), below $55 K (May et al., 2012), over $100 K (Narcisa et al., 2013), or over $50 K (Xu et al., 2018). Most participants based in Canada had an income between $20–80 K (Regier et al., 2009), while most Taiwanese participants had an income between $20–40 K (Chen et al., 2020). Finally, two studies provided a summary of participants’ socioeconomic level qualifying it as “median of 5 in a scale of 1 to 10” (Hendel et al., 2021) or “varied including families living in poverty and families in top earning categories” (Navot et al., 2016).

The participants’ employment status was reported in 12 studies, again in the form of number or percentage of parents reporting each employment category or in one study as the mean number of working parents (Hendel et al., 2021). Across these studies, the most frequently reported category was “employed” or “employed full-time” (n = 1166), followed by homemakers or unemployed (n = 567; based on data from eight studies reporting participant numbers; Chen et al., 2015a, 2015b; Giarelli & Reiff, 2015; Li et al., 2022; Selkirk et al., 2009; Xu et al., 2016; Zhao et al., 2018, 2019a, 2019b).

The participants’ spoken language was reported in 18 studies, with six studies reporting more than one language. English was the predominant language, reported in 14 studies, followed by Chinese/Mandarin (n = 4; Chen et al., 2013; Chen et al., 2020; Li et al., 2022; Zhang et al., 2021), French (n = 1; Amiet et al., 2014), Turkish (n = 1; Ayhan et al., 2021), Hebrew (n = 1; Hendel et al., 2021), and Taiwanese (n = 1; Chen et al., 2013).

Data on marital status were reported in 14 studies, again as a number or percentage of parents reporting each category. The most frequently reported marital status was being/living as married (n = 1629 participants, followed by one-parent households including divorced/separated/widowed/widower (n = 188), and never married/single/other (n = 121).

When examining family history of ASD, defined as having at least one additional member of the close or extended family diagnosed with ASD, excluding the participant’s own child, data from seven studies revealed that most participants had no family history of ASD (n = 1100), followed by participants with a family history of ASD (n = 600), and those unsure about their family history of ASD (n = 102). Studies reporting percentages rather than numbers corroborated that “no family history of ASD” was the most reported category.

### Children Characteristics

All 30 studies reported data on the children’s diagnosis, with 18 stating that this was ASD or autism without further details (Supplementary Information). In seven studies, the children’s diagnosis was reported as the exact number of participants belonging to each diagnostic category, with autistic disorder, ASD or pervasive development disorder not otherwise specified (PDD-NOS) being the predominant ones (Amiet et al., 2014; Cleary et al., 2022; Li et al., 2022; Petruccelli et al., 2022; Selkirk et al., 2009; Wagner et al., 2020; Zhao et al., 2019a). In the three studies reporting percentages, again, ASD and autistic disorder were the predominant diagnoses (Narcisa et al., 2013; Zhao et al., 2019b, 2021a, 2021b). In two studies, the diagnosis was reported as “developmental disability” (Regier et al., 2009) or “autistic disorder or PDD-NOS” (Navot et al., 2016) without any further details. Most children whose parents participated in reported studies had ASD or autism (n = 969), followed by PPD-NOS (n = 576) and Asperger’s syndrome (n = 423). The rest of the children were reported as typically developing (n = 80), with a developmental delay (n = 39, with more than two disorders (n = 22), with a language impairment (n = 4), with a global developmental delay (n = 1), or with an unknown/uncertain diagnosis (n = 150).

The children’s age at the time that the study was conducted was reported in half of the studies only (n = 15), in the form of a mean age (n = 11) or an age range (n = 4). The most commonly reported mean ages were over 10 years (n = 5Selkirk et al., 2009; Zhao et al., 2018, 2019a, 2019b, 2021a, 2021b); between 0 and 5 years (n = 3; Cleary et al., 2022; Lemay et al., 2021; Wagner et al., 2020), or between 5 and 10 years (n = 3; Chen et al., 2020; Giarelli & Reiff, 2015; Xu et al., 2018). Studies reporting an age range pointed to 3 to 6 years being the most common (Ayhan et al., 2021; Reiff et al., 2015) or 14 to 59 months (Navot et al., 2016; Petruccelli et al., 2022).

The children’s age when screening for ASD was conducted was reported in only four studies. Giarelli and Reiff (2015) reported that their participants’ mean age when they underwent genetic testing was 7.6 years. Ayhan et al. (2021) reported that the most common age range for genetic testing was between 2 and 3 years (n = 678), followed by 4 to 5 years (n = 174), and 0 to 1 year (n = 69). Reiff et al. (2015) reported 5–12 months as the most common age range of genetic testing (n = 27), followed by over 25 months (n = 15), 13–18 months (n = 8), and 19–24 (n = 4). Finally, Petrucelli et al. (2022) reported 18 to 24 months as the most commonly reported age range for behavioral screening without providing any further details.

To examine the number of siblings with ASD, we were able to extract data from 15 studies. Most participants reported having an only child with ASD, followed by two children with ASD or more than two siblings with ASD. Based on data extracted from the 11 studies that reported the exact numbers of children with ASD in the family, 3172 children were an only child with ASD, 143 children with ASD had one sibling who also had ASD, and 196 children with ASD had two or more siblings with ASD (Amiet et al., 2014; Ayhan et al., 2021; Chen et al., 2015a, 2015b; Cleary et al., 2022; Hendel et al., 2021; Li et al., 2022; Navot et al., 2016; Selkirk et al., 2009; Zhao et al., 2018, 2019a, 2019b).

### Study Characteristics

Of included studies, more than half utilized quantitative methods (n = 16), one third used qualitative methods (n = 10; Chen et al., 2015a, 2015b; Chen et al., 2015a, 2015b; Chen et al., 2013; Cleary et al., 2022; Giarelli & Reiff, 2015; Navot et al., 2016; Petruccelli et al., 2022; Xu et al., 2016; Zhang et al., 2021; Zhao et al., 2018), and four employed mixed methods (Lemay et al., 2021; Reiff et al., 2015, 2017; Selkirk et al., 2009).

**Fig. 3:**
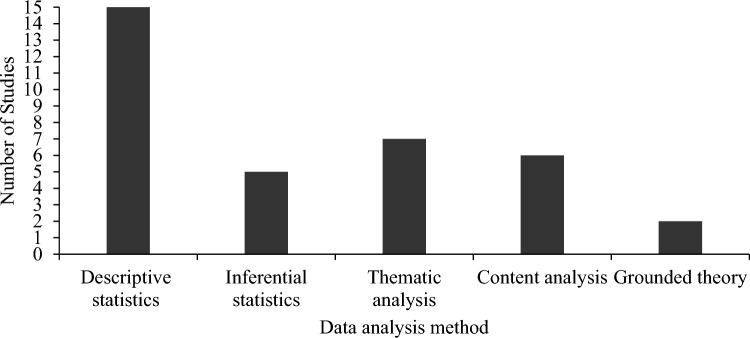
Data analysis method used across studies

Data were collected via a variety of modalities (Fig. 3), with seven studies using two different modalities (Ayhan et al., 2021; Chen et al., 2020; Lemay et al., 2021; Lucas et al., 2022; Petruccelli et al., 2022; Reiff et al., 2017; Xu et al., 2018), and two studies using three (Chen et al., 2013; Reiff et al., 2015). The most common data collection modality reported was online surveys (n = 15), followed by in-person interviews (n = 14), phone calls (n = 7), paper and pencil surveys sent by post (n = 4), and video calls (n = 1).

### Type of Early Screening

In the vast majority of studies participants reported having undergone genetic testing alone (n = 26), with participants in the four remaining studies reporting that their child underwent early life behavioral screening alone (Lemay et al., 2021; Petruccelli et al., 2022) or both genetic and behavioral screening (Cleary et al., 2022; Navot et al., 2016). The type of genetic testing was reported in 14 studies as a chromosomal microarray analysis (CMA; n = 7), prenatal genetic testing (PGT; n = 3), Fragile-X (n = 2), Monoamine oxidase A related to child aggression (MAO-A; n = 1), array genomic hybridization (AGH; n = 1), single gene or gene panel testing (n = 1), or whole exome sequencing (WES; n = 1).

### Data Analysis

In half of the studies, data were analyzed using descriptive statistics alone (n = 12) or combined with another data analysis method (n = 3; Lemay et al., 2021; Selkirk et al., 2009; Zhao et al., 2019b). Inferential statistics were used in five studies, either alone (May et al., 2012; Regier et al., 2009; Wagner et al., 2020) or in combination with another data analysis method (Reiff et al., 2015, 2017). Thematic analysis was used alone in four studies (Cleary et al., 2022; Giarelli & Reiff, 2015; Selkirk et al., 2009; Xu et al., 2016) and in combination with another data analysis method in three studies (Lemay et al., 2021; Reiff et al., 2015, 2017). Content analysis was used in four studies as a standalone method (Chen et al., ; Zhang et al., 2021) and in two studies in combination with another method (Selkirk et al., 2009; Zhao et al., 2019b). Finally, grounded theory was used in two studies (Navot et al., 2016; Petruccelli et al., 2022). Overall, five studies used a combination of two data analysis methods (Lemay et al., 2021; Reiff et al., 2015, 2017; Selkirk et al., 2009; Zhao et al., 2019b). Figure 3 shows the breadth of data analysis methods employed across the 30 studies.

### Emerging Themes

Data collected on parental perspectives were grouped into two categories: benefits and barriers. We identified a total of 20 emerging themes across the 30 studies, with 13 of these representing positive attitudes or benefits (Table 2) and seven representing negative attitudes or barriers (Table 3).

**Table 2:** Emerging themes on benefits of genetic testing

	Amiet et al. (2014)	Ayhan et al. (2021)	Chen et al. (2013)	Chen et al. (2015b)	Chen et al. (2015a)	Chen et al. (2020)	Cleary et al. (2022)	Girarelli & Reiff (2015)	Hendel et al. (2021)	Lemay et al. (2021)	Li et al. (2022)	Lucas et al. (2022)	May et al. (2012)	Narcisa et al. (2013)	Navot et al. (2016)	Petruccelli et al. (2022)	Reiff et al. (2015)	Reiff et al. (2017)	Reiger et al. (2009)	Selkirk et al. (2009)	Wagner et al. (2020)	Xu et al. (2016)	Xu et al. (2018)	Zebolsky et al. (2020)	Zhang et al. (2021)	Zhao et al. (2018)	Zhao et al. (2019a)	Zhao et al. (2019b)	Zhao et al. (2021a)	Zhao et al. (2021b)
Knowledge on ASD	●	●	●		●		●	●	●	●	●	●		●			●		●	●	●	●	●	●			●	●	●	●
Family planning		●	●	●	●	●	●		●			●			●		●			●	●	●	●	●	●	●		●		●
Accessing medical treatment			●	●				●				●	●	●		●	●	●		●	●		●	●	●	●	●	●		●
Etiology of ASD/ Family history			●		●	●		●	●		●				●			●		●	●	●	●	●	●	●		●	●	●
Accessing early intervention	●		●				●			●		●	●	●		●	●	●		●	●	●		●	●	●	●	●		
Early diagnosis/ detection	●	●				●	●			●		●		●			●	●		●		●	●		●	●		●		●
Counselling/emotional support							●	●		●		●		●	●	●	●					●						●	●	●
Family support			●				●			●		●			●	●	●						●						●	●
Preparation for childbirth				●		●						●	●					●				●						●		
ASD severity				●		●					●		●		●														●	●
Terminate pregnancy				●		●																		●		●

**Table 3:** Emerging themes on barriers to genetic testing

	Amiet et al. (2014)	Ayhan et al. (2021)	Chen et al. (2013)	Chen et al. (2015b)	Chen et al. (2015a)	Chen et al. (2020)	Cleary et al. (2022)	Girarelli & Reiff (2015)	Hendel et al. (2021)	Lemay et al. (2021)	Li et al. (2022)	Lucas et al. (2022)	May et al. (2012)	Narcisa et al. (2013)	Navot et al. (2016)	Petruccelli et al. (2022)	Reiff et al. (2015)	Reiff et al. (2017)	Reiger et al. (2009)	Selkirk et al. (2009)	Wagner et al. (2020)	Xu et al. (2016)	Xu et al. (2018)	Zebolsky et al. (2020)	Zhang et al. (2021)	Zhao et al. (2018)	Zhao et al. (2019a)	Zhao et al. (2019b)	Zhao et al. (2021a)	Zhao et al. (2021b)
Lack of health providers recommendations	●		●		●	●		●	●					●	●		●	●		●	●	●		●	●	●	●	●	●	●
Cost				●							●		●			●			●		●	●	●	●	●		●	●	●	●
Genetic testing accuracy				●				●					●							●	●		●	●	●		●	●		●
Cultural/religious beliefs			●	●	●	●		●	●		●				●	●								●		●				
Potential harmful side effects of testing			●			●		●														●	●	●	●	●			●	●
Low education/ income	●	●									●			●					●		●		●				●		●	
Discrimination		●			●	●			●				●	●		●														●
Privacy concerns		●						●	●												●									

Potential benefits of genetic testing for autism included identifying the possible cause of autism including the presence of family history of ASD, promoting early detection and intervention, determining the severity of ASD, helping develop treatment plans that target autism-related medical conditions, and facilitating risk assessments that could lead to counselling, emotional support, and education for parents. The majority of the participants thought that genetic testing would be beneficial for their child, their reproductive choices, family and childbirth planning, potential future generations, and ASD research. Participants stated that if a test reveals a harmful mutation with known ties to autism, the result could give the person with autism and their family an explanation for the condition. Some participants also reported having found emotional and practical support from others dealing with the same mutation. Recurring benefits can be seen in Table 2, ordered by frequency of appearance from most to least cited theme.

Identified barriers for parents to access genetic testing for ASD included the cost of the genetic tests, parents not knowing whether the results would yield accurate results, the lack of recommendations from health providers, discrimination towards the family of the child with ASD, cultural and religious beliefs, and privacy concerns about results data storage. Participants stated that many providers choose not to offer testing to families and many families are unaware of the option of genetic testing. Also, participants reported concerns related to possible harmful side effects of genetic testing (Table 3; recurring barriers ordered from most to least frequently cited).

From coding results in positive, negative, or mixed parental attitudes towards genetic testing, we identified four studies as positive and 26 reporting mixed results. None of the studies was classified as negative.

## Discussion

Our systematic review aimed to identify studies examining parental perspectives around early-life genetic screening for autism. We also made a deliberate attempt to categorize findings into meaningful themes that will guide future research and practice. Over the past 15 years, there has been a steady increase in studies on early childhood ASD screening (before 24 months). These studies, initially concentrated in the US and Canada, now encompass diverse global regions such as Taiwan, Australia, and France. This trend underscores an expanding research interest in this area and heightened awareness of ASD early life screening on a worldwide scale. Previous reviews focusing on parental views around ASD diagnosis have highlighted the effect of personal, cultural, and environmental factors (Makino et al., 2021; Naicker et al., 2023), and our review offers a novel insight into the effect of such factors on parental views on early genetic screening.

The prevalent demographic of participating parents was notably specific: US-based English speaking white female, aged 35 to 45, Christian, with university degree, employed full-time, with income over $75 K, married, with at least one child with ASD, and without any prior family history of ASD. The most commonly described child profile was being the only child with ASD and aged 2–3 years when screening for ASD was conducted.

Our review also serves as a benchmark for future studies synthesizing research methods, data collection strategies, and critical parent and child characteristics. Our findings suggest that researchers predominantly used quantitative research methods, with data collected primarily through online surveys, which may be due to the ability of online surveys to reach a larger sample. For instance, Ayhan et al. (2021) engaged 951 participants, while Zhao et al. (2019a, 2019b) surveyed 552 individuals. The preference for online surveys might stem from their efficiency and the fact that they can reach key stakeholders residing outside urban centers or far from research facilities. Notably, very few studies deviated from this predominant trend. For example, Chen et al. (2013) employed a combination of video calls, phone calls, and in-person interactions, accommodating a modest sample size of 42 participants within a qualitative research framework. It is worth highlighting that Chen et al. used video calls for a subset of five participants within their study. Reiff et al. (2015, 2017) also employed three data collection modalities within a mixed methods approach. The limited use of video calls across studies may be attributed to the potential reluctance on the part of participating families to engage in this data collection modality or to the fact that researchers only offered in-person or phone calls as data collection methods, which had been the norm before the pandemic. Future researchers may consider the feasibility and acceptability of using technology to reach participants when employing data collection methods other than online surveys (e.g., using video calls to conduct focus groups or interviews in qualitative research).

Parents reported both behavioral screening tools and genetic tests when examining early-life screening methods. Geneticists have documented a total of 103 genes and 44 genomic loci as linked to ASD. For some of the identified genes and genomic loci, researchers have established a robust correlation with an ASD phenotype (Betancur, 2011). Moreover, for more than a decade, clinical geneticists have been offering ASD genetic testing as part of medical practice (Schaefer & Mendelsohn, 2013). Notably, our findings align with the conclusions drawn by Schaefer and Mendelsohn (2013), who described Chromosomal Microarray Analysis (CMA) as a potent tool for clinical ASD testing, given there was a consensus among eligible studies that CMA was the most frequently utilized method of ASD genetic testing.

To draw meaningful conclusions on parental perspectives around early life genetic testing for ASD, we categorized emerging themes into benefits and barriers. A total of 20 recurring themes emerged, with 13 representing benefits and seven representing barriers. Overall, parents were positively disposed towards genetic testing, stating that it could explain the cause of ASD, assist in family planning, and facilitate early access to intervention, among numerous other benefits. The most commonly cited benefits were increasing knowledge about ASD, having an explanation for its cause, and accessing medical treatment. Reported barriers included lack of information from health providers, the cost of genetic testing, uncertainty around its accuracy, potential side effects of a genetic test, discrimination, data privacy concerns, and cultural or religious beliefs not being aligned with genetic testing, among others. The two barriers cited most often were the lack of information about genetic testing and its cost. These findings highlight the need to offer training to healthcare providers in line with parental requirements for effective communication around genetic testing for ASD and counseling services that can empower parents to make informed decisions. Public funding and private insurance schemes can mitigate the cost barrier and reduce disparities.

From these results, it becomes apparent that health providers should augment parental awareness of ASD and ASD genetic testing and provide clear recommendations on how to access genetic testing, should they wish to do so. Despite these themes emerging in distinct cultural contexts, collectively findings underscore the desire of families to be well-informed about existing screening procedures with an underlying belief that their healthcare providers should provide such information. This finding aligns with recent reviews on ASD diagnosis (Makino et al., 2021; McCrimmon & Gray, 2021; Naicker et al., 2023). Regardless of geographical location, families share the common sentiment of feeling ill-prepared to make decisions around ASD genetic testing. Future research should explore strategies for enhancing healthcare professionals’ knowledge in this area and determining how they can proactively inform families of ASD genetic testing, including data privacy and consent aspects, even when this is not explicitly requested. Because parents strongly desire to know more about genetic testing, it is reasonable to assume that families not asking for information during initial contact with healthcare providers might not do so due to a lack of awareness rather than a lack of interest or positive predisposition. More importantly, genetic testing taken early in life might confirm existing developmental concerns, such as those often present in younger siblings of a child with ASD, enabling access to pre-emptive behavioral intervention when brain plasticity is at its peak (Tanner & Dounavi, 2020). Emerging genetic tests are likely to increase diagnostic accuracy for ASD in the near future. This underscores the need for research to focus on implementing these tests in ways that meet parental needs and preferences.

Several limitations are worth noting in the context of studies included in this systematic review. Despite the application of rigorous inclusion criteria, it is essential to acknowledge the variability in the quality of the selected studies. Such variability may stem from limitations in sample size, research design, or data collection methods, which, in turn, have the potential to influence the overall robustness of synthesized evidence. Future research should adopt a mixed methods approach that combines high-quality quantitative and qualitative methods, allowing researchers to provide an in-depth understanding of parental viewpoints. Moreover, a bias is evident within the included studies, as our findings rely on studies predominantly conducted in the US with White female participants of a high socioeconomic status. Such bias restricts the generality of our findings from a translational perspective. Culture plays a pivotal role in shaping attitudes. Research failing to encompass the views and preferences of a culturally diverse population is at a high risk of low uptake by the community it is set to serve. Future research should examine whether the present findings remain valid across cultures by engaging a broader range of populations, encompassing diversity in location, gender, age, cultural background, and socioeconomic status. To meet this aim, specific outreach to the communities under-represented in our review should be prioritized. This could be facilitated by adopting a multicultural approach to research, e.g., by involving researchers and clinicians from diverse backgrounds and locations and using multilingual parental surveys.

In sum, our review shows that parents wish to access genetic testing with their views being profoundly shaped by their desire to understand ASD and seek medical treatment and intervention as early as possible. Healthcare providers can be pivotal in empowering parents to make informed decisions through clear, compassionate communication and counseling. Future research should seek to fill in an essential gap in the literature, which is to capture parental views from a diverse population.
